# Unusual Perceptual Experiences at the End-of-Life: Caregiver Responses and Implications for Symptom Management

**DOI:** 10.1093/geroni/igaf122.4229

**Published:** 2025-12-31

**Authors:** Kelly Melekis, Elizabeth Cepeda, Carol Weisse, Meredith Killian

**Affiliations:** University of Vermont, Burlington, Vermont, United States; University of Vermont, Burlington, Vermont, United States; Union College, Schenectady, New York, United States; Union College, Schenectady, New York, United States

## Abstract

Unusual perceptual experiences (UPEs) occurring near death are a universal human phenomenon, with accounts recorded across time and cultures (Kellehear, 2016). For those on hospice, characterizations of UPEs and levels of both patient and caregiver distress may influence decisions about symptom management. The predominance of the routine home care setting rather than an institutional or hospitalized setting highlights the importance of understanding how informal caregivers manage UPEs in these settings, especially in light of research showing that medication administration for managing EOL symptoms varies by setting (Arevalo et al., 2018). This study utilized a mixed-method retrospective record review of 138 deceased hospice patients to better understand the presence and characterization of UPEs at EOL, caregiver perceptions of symptoms, and symptom management in home care settings. 36% of patients were identified as having > 1 UPE. While the majority (51%) experienced a single UPE, there were a total of 137 instances among 49 patients. Distribution of PRN psychotropic medication following a UPE incident was indicated only 14% of the time. The vast majority never received an antipsychotic PRN (88%) or a benzodiazepine PRN (80%) following a UPE incident, and there was little to no documentation of behavioral interventions for symptom management. The variability with which PRN medication was administered suggests caregivers may be uncertain about when to administer medications. Study results point to a need for enhanced caregiver training on strategies for assessing and documenting UPEs, and decision-making for symptom management, including both pharmacologic and behavioral interventions.

